# Supplementary material for: The interactome of intact mitochondria by cross-linking mass spectrometry provides evidence for coexisting respiratory supercomplexes
Source: Mol Cell Proteomics. 2017 Dec 8;17(2):216–32. doi: 10.1074/mcp.RA117.000470 (PMC5795388; doi:10.1074/mcp.RA117.000470)
Supplement: Supplemental Data [file supp_RA117.000470_133922_0_supp_23978_fzffwf.zip › spectra_annotation/mito_spectra_annotation/66-1-10-1-6-1.pdf]

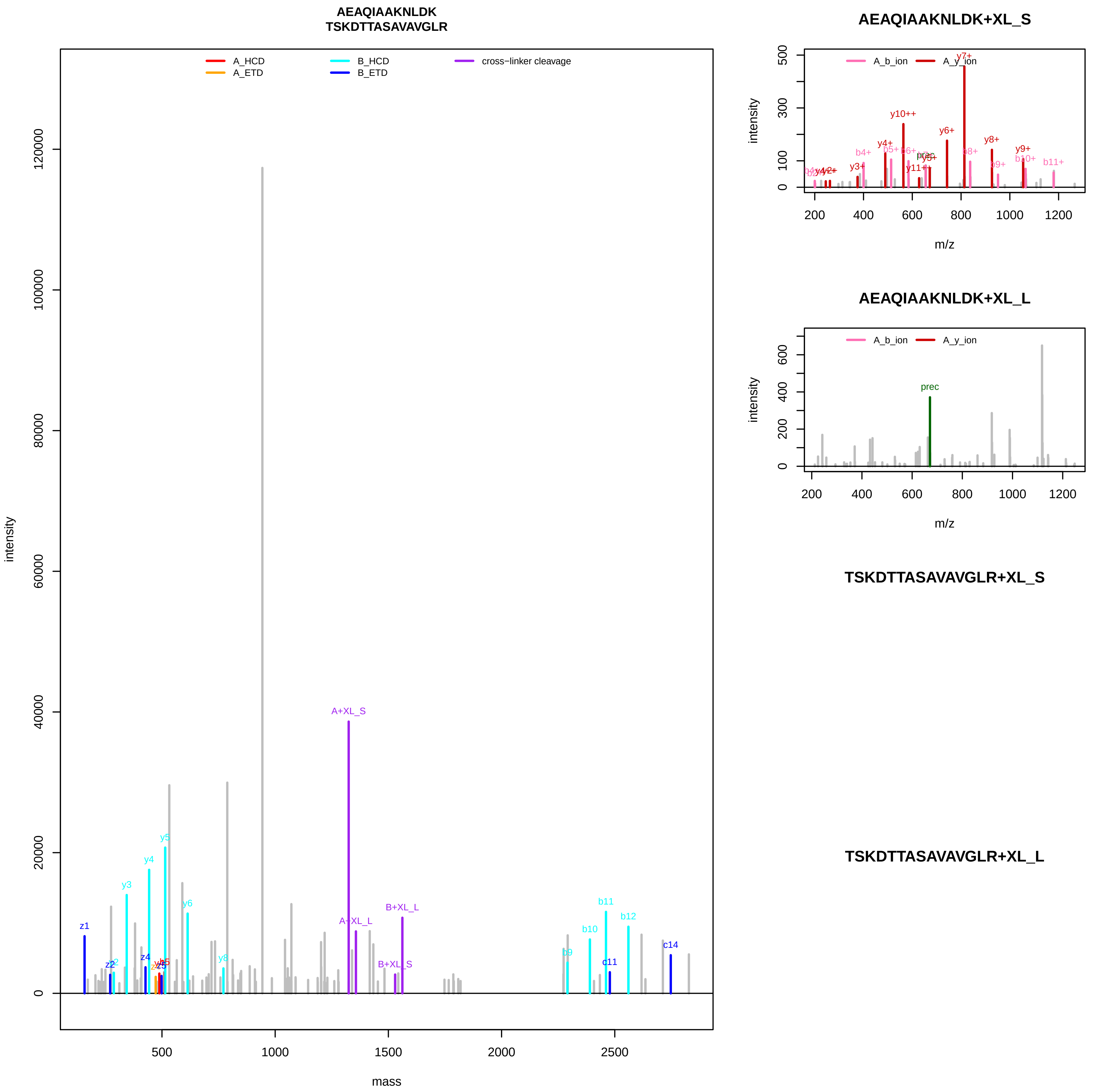
AEAQIAAKNLDK
TSKDTTASAVAVGLR
A_HCD
A_ETD
B_HCD
B_ETD
cross−linker cleavage
intensity
mass
A+XL_S
A+XL_L
B+XL_L
B+XL_S
z1
z2
y3
y4
z4
y5
y6
y8
b9
b10
b11
c11
b12
c14
AEAQIAAKNLDK+XL_S
A_b_ion
A_y_ion
y10++
y6+
y7+
y8+
y9+
y4+
y3+
b4+
b5+
b6+
y11++
b8+
b9+
b10+
b11+
prec
m/z
AEAQIAAKNLDK+XL_L
TSKDTTASAVAVGLR+XL_S
TSKDTTASAVAVGLR+XL_L
